# Additional Commentary on “The Proposed 48-Month Emergency Medicine Residency Requirement Demands Immediate Scrutiny”

**DOI:** 10.5811/westjem.48652

**Published:** 2025-06-25

**Authors:** Stephen Hayden

**Affiliations:** University of California, San Diego, Department of Emergency Medicine, Southwest Medical Education Consortium Emergency Medicine Residency, San Diego, California

## Abstract

This paper provides commentary on the accompanying publication, “The Proposed 48-Month Emergency Medicine Residency Requirement Demands Immediate Scrutiny.” The ACGME Residency Review Committee for Emergency Medicine recently proposed a change to the required length of training to 48 months. Currently, there is a lack of objective data to support the optimal duration of emergency medicine residency training. One of the primary concerns regarding a mandated fourth year is the significant financial burden it would place on training programs. If sponsoring institutions are unable or unwilling to provide the necessary resources to support a prolonged curriculum, programs could be compelled to reduce resident class sizes. A reduction in class size would negatively impact the educational environment, including emergency department coverage and participation in external rotations. To better prepare physicians for independent practice, it may be time to consider a base training length of 36 months, followed by alternative pathways such as fellowships, focused practice designations, or targeted curricula—all of which may be more effective than extending the duration of residency training.

The debate regarding the optimal duration and format for emergency medicine (EM) residency training has persisted for over three decades among program directors (PD) and professional organizations. Historically, discussions among PDs from both three- and four-year programs have often ended with a reluctant consensus: that three and a half years might be a reasonable compromise. This conclusion, however, was based largely on anecdotal experience. Some residents demonstrated readiness for independent practice at the end of three years, while others benefited from a fourth year. At the time, objective data were scarce, and no single organization had assumed the responsibility of establishing a standardized model for EM training.

As a result, individual programs—along with their PDs, sponsoring institutions, and affiliated clinical sites—have independently determined their educational goals, curricular designs, and resource allocations to be compliant with the requirements of the Accreditation Council for Graduate Medical Education. Although this approach lacks uniformity, it has largely served the specialty well. Over the past three decades, emergency departments (ED) have faced a growing array of clinical and operational challenges, including emerging diseases, evolving care paradigms, rising patient volumes, and increasing length of ED boarding times. Despite these mounting demands, most EM residency programs have continued to produce competent, practice-ready physicians while successfully integrating new technologies, managing public health crises, and addressing administrative responsibilities.

For the sake of transparency, the authors of this commentary collectively possess over 75 years of experience in EM education, curriculum development, clinical teaching, and training standard design. We have directed both three- and four-year programs and are well-versed in the respective strengths and limitations of each format. Our leadership roles span Program Director, Education Dean, Designated Institutional Official, and officer positions within the Council of Residency Directors in Emergency Medicine (CORD), including a past presidency. While it is reasonable to assume that members of the Program Requirements Writing Group and summit attendees also have considerable experience, the omission of detailed qualifications in the methodology section weakens the transparency and credibility of the report.

One of the central arguments against a mandated fourth year of residency is the significant financial burden it would impose. Beyond compounding medical student debt, an additional year of training would likely strain institutional budgets. Although the Centers for Medicare & Medicaid Services (CMS) has indicated that longer EM training would proportionally increase federal funding for graduate medical education, many programs rely only partially on CMS support and depend on other, often limited, funding sources. If sponsoring institutions are unable or unwilling to provide additional resources, programs may be forced to reduce class sizes to accommodate a longer curriculum. This would not only be a financial concern but also an educational one.

Reduced class size could negatively affect residents’ learning environments, including ED coverage and engagement in external rotations. For instance, when EM residents rotate consistently on trauma services, they become integral members of the team. This continuity fosters trust, increases access to procedures, and enhances learning opportunities. If fewer residents are available due to smaller class sizes, such continuity and integration may be compromised, diminishing the educational quality of these experiences. Although difficult to quantify, these impacts are nonetheless meaningful.

While it is true that the scope of EM is expanding into increasingly diverse and specialized areas, this evolution may call for a modified, competency-based approach to training. Rather than adhering to the traditional “Anyone, Anything, Any time” mantra, a more practical goal for core EM education would be to prepare physicians for safe, autonomous practice in the most common clinical settings. Training for more specialized environments—such as rural, austere, academic, or military practice—could be pursued through post-residency fellowships, targeted curricula for added qualifications or focused practice designations.

This model mirrors the pathway established in general surgery, where completion of a core residency may be followed by subspecialty training in acute care surgery, accredited by the American Association for the Surgery of Trauma. Similarly, EM training could consist of a standardized 36-month core curriculum, followed by an optional 12-month (or longer) period for individualized training in areas such as ultrasound, administration, global health, or critical care. This structure would preserve flexibility for both residents and residencies and allow programs to maintain their current duration of training while ensuring rigorous preparation for board certification and independent practice. Moreover, providing options for extended training would likely support recruitment by offering applicants greater alignment between program offerings and personal career goals. Our specialty is constantly evolving to meet the demands of a changing healthcare environment, so should our approach to educating our residents and determining their training requirements.

Link to Original Commentary: https://escholarship.org/uc/item/51w1011s

